# Prolactin Is a Strong Candidate for the Regulation of Luteal Steroidogenesis in Vizcachas (*Lagostomus maximus*)

**DOI:** 10.1155/2018/1910672

**Published:** 2018-06-14

**Authors:** S. Proietto, S. A. Cortasa, M. C. Corso, P. I. F. Inserra, S. E. Charif, A. R. Schmidt, N. P. Di Giorgio, V. Lux-Lantos, A. D. Vitullo, V. B. Dorfman, J. Halperin

**Affiliations:** ^1^Centro de Estudios Biomédicos, Biotecnológicos, Ambientales y Diagnóstico (CEBBAD), Universidad Maimónides, Ciudad Autónoma de Buenos Aires, Argentina; ^2^Consejo Nacional de Investigaciones Científicas y Técnicas (CONICET), Buenos Aires, Argentina; ^3^Laboratorio de Neuroendocrinología, Instituto de Biología y Medicina Experimental (IByME), Ciudad Autónoma de Buenos Aires, Argentina

## Abstract

Prolactin (PRL) is essential for the maintenance of the corpora lutea and the production of progesterone (P4) during gestation of mice and rats, which makes it a key factor for their successful reproduction. Unlike these rodents and the vast majority of mammals, female vizcachas (*Lagostomus maximus*) have a peculiar reproductive biology characterized by an ovulatory event during pregnancy that generates secondary corpora lutea with a consequent increment of the circulating P4. We found that, although the expression of pituitary PRL increased steadily during pregnancy, its ovarian receptor (PRLR) reached its maximum in midpregnancy and drastically decreased at term pregnancy. The luteinizing hormone receptor (LHR) exhibited a similar profile than PRLR. Maximum P4 and LH blood levels were recorded at midpregnancy as well. Remarkably, the P4-sinthesizing enzyme 3*β*-HSD accompanied the expression pattern of PRLR/LHR throughout gestation. Instead, the luteolytic enzyme 20*α*-HSD showed low expression at early and midpregnancy, but reached its maximum at the end of gestation, when PRLR/LHR/3*ß*-HSD expressions and circulating P4 were minimal. In conclusion, both the PRLR and LHR expressions in the ovary would define the success of gestation in vizcachas by modulating the levels of 20*α*-HSD and 3*ß*-HSD, which ultimately determine the level of serum P4 throughout gestation.

## 1. Introduction

The corpus luteum (CL) is a transient endocrine gland that biosynthesizes steroids under the control of luteotropic factors. The CL synthesizes large amounts of progesterone (P4), which has an important role in the modulation of the estrous cycle and in the maintenance of pregnancy as well as an intermediary role in the synthesis of corticosteroids and androgens ([1–3] for a review). In mice and rats, the corpora lutea are the main source of P4 that will support the gestation process. In fact, mouse models in which the P4 receptor (*PR*) has been ablated are infertile and display reproductive abnormalities as embryo implantation failure, defects in uterine decidualization, and an abnormal response to estradiol, thus demonstrating how critical P4 is for the normal progress of pregnancy [4–6]. P4 exerts its action via the PR, a member of the nuclear receptor superfamily of transcription factors. Upon ligand binding, the PR dimerizes and binds to PR response elements in the promoters of its target genes such as bone morphogenetic protein 2 (*Bmp2*), homeobox A10 (*Hoxa10*), and Indian hedgehog (*Ihh*), all molecules whose important actions during gestation have already been widely established ([7], for a review).

The CL expresses key proteins and steroidogenic enzymes involved in the uptake, synthesis, and transport of cholesterol and in the processing of cholesterol to P4. The first step in the path of P4 biosynthesis is performed by the cytochrome P450 side-chain cleavage (P450scc), which converts cholesterol into pregnenolone. In turn, pregnenolone is converted into the active metabolite, P4, by the action of the oxidoreductase enzyme 3*ß*-hydroxysteroid dehydrogenase/*Δ* ^5^-Δ^4^ isomerase (3*β*-HSD) [8]. P4 production is tied to the expression level of these two enzymes. In fact, Richards and coworkers have documented a significant increase both in P450scc and 3*β*-HSD transcript levels in luteal cells during the luteinization process [9, 10].

On the contrary, 20*α*-hydroxysteroid dehydrogenase (20*α*-HSD) catalyzes the reduction of P4, leading to the formation of the progestationally inactive steroid, 20*α*-hydroxypregn-4-en-3-one (20*α*-hydroxyprogesterone). This enzyme also belongs to the family of oxidoreductases, and it has shown to play a significant role in luteolysis and parturition. While luteal 20*α*-HSD expression and activity are downregulated in mice and rats, 24 h prior to parturition ovarian 20*α*-HSD activity is acutely stimulated. Such stimulation is mandatory for the final reduction of P4 blood levels preceding parturition [11–16]. These two enzymes that participate in the metabolism of P4 are targets of tropic hormones such as prolactin (PRL) and luteinizing hormone (LH), as well as of endogenous and circulating steroids [3, 17].

Prolactin (PRL) is a 23 kDa protein mainly secreted by lactotropic cells of the anterior pituitary gland, and it has been originally identified by its stimulatory action on mammary glands during pregnancy and lactation. However, it is now known that besides the mammary glands, PRL targets several other tissues expressing its membrane-bound receptor (PRLR) and modulates a great variety of biological functions ([18] for a review). Focusing on the implications of PRL actions over the reproductive process, it has been shown in transgenic mice models that ablation of either *PRL* or *PRLR* gene greatly impairs female fertility [19, 20]. The lack of PRL signaling leads to the impossibility of maintaining the implantation of the embryo mainly due to an insufficient production of luteal P4. Two days after mating, *PRLR* knockout ovaries exhibit corpora lutea undergoing regression and an impairment in steroidogenesis [21]. This clearly establishes the critical role of PRL in the maintenance of the CL and production of P4 for rodent reproduction ([2, 3, 22, 23] for a review).

The South American plains vizcacha, *Lagostomus maximus* (Rodentia: Chinchillidae), is a hystricognathe fossorial rodent that inhabits the Pampas region of Argentina extending up to the South of Paraguay and Bolivia [24]. Over forty years ago, Barbara Weir described particular aspects of the reproductive biology of female vizcachas that highlight this species among the majority of mammals [25, 26]. The ovaries of adult specimens exhibit natural massive polyovulation that can go up to 800 oocytes per cycle, the highest ovulatory rate so far recorded for a mammal. Although the oocytes are ovulated literally in hundreds, just 10 to 12 of them will be eventually fertilized and implanted, but only those two implanted nearest the cervix will complete their embryonic development [26, 27]. An unusual long gestation is also a distinctive trait of vizcachas. Its 5-month length pregnancy is considered a long period for a rodent, and it is one of the longest recorded among hystricomorphs [25].

But what undoubtedly is the most outstanding aspect of its reproductive profile is the atypical gestational hormonal pattern characterized by the release of gonadotropin-releasing hormone (GnRH) followed by an LH surge around day 110 of gestation. This rise in LH leads to an ovulatory event that produces a great number of secondary corpora lutea with oocyte retention (i.e., pseudoovulation) and a consequent and marked increment of the P4 levels [28–31]. This uncommon boost up in the circulating P4 may contribute to the accurate maintenance of the uterus and embryo development up to the end of a long gestation [28, 32]. Since PRL has shown to be essential for synthesis and secretion of luteal P4 during pregnancy in both mice and rats, we hypothesized that PRL also plays a role in the luteal steroidogenesis in pregnant vizcachas. For that, we examined ovarian PRLR expression as well as that of several P4 production-modulating molecules and we found a consistent expression pattern along gestation for ovarian PRLR/LHR/3*β*-HSD that contrasts with that of 20*α*-HSD, pinpointing PRL and LH as central players in the modulation of luteal steroidogenesis and P4 production in pregnant vizcachas.

## 2. Material and Methods

### 2.1. Animal Handling

Twenty-five female vizcachas, *L. maximus*, weighting between 1.9 and 2.5 kg were used throughout the present study. Animals were captured in their habitat using live traps placed in their burrows in a natural population site at the *Estación de Cría de Animales Silvestres* (ECAS), Province of Buenos Aires, Argentina (34° 51′ 0^″^ S, 58° 6′ 37^″^ W). The capture and transport of animals were approved by the Ministry of Agriculture Authority of the Buenos Aires Province Government. Animals were housed for one week before euthanasia, under a 12 : 12 h light cycle to simulate their natural luminal exposure (low light of 12 watts followed by moon light) and 22 ± 2°C room temperature, with ad libitum access to food and tap water.

Animals were grouped according to their reproductive status as the following: early pregnancy (EP), midpregnancy (MP), and term pregnancy (TP), lactating nonpregnant (LCT) and non-ovulating non-pregnant (NP) females (Table 1). In order to establish the different groups, the time of capture was carefully planned according to the natural reproductive cycle of the vizcachas previously described by Llanos and Crespo [33] and also based on our own field expertise [28–32, 34–36]. Pregnant vizcachas were captured during the breeding season that lasts from April to August. Gestational stage was estimated by the time of capture and confirmed during the surgical procedure by the degree of fetal development as previously described [32, 37]. The midpregnant group was formed with pregnant individuals whose ovaries exhibited, at the time of sacrifice, ovulatory stigmatas as evidence of a recent ovulatory event and later confirmed for the presence of secondary corpora lutea in hematoxylin-eosin stained ovary sections. Nonpregnant females were captured in mid-September after the end of the reproductive season. Lactating female group (LCT) was established by selecting those nonpregnant animals whose mammary glands exhibited the histological features already described for this period [29, 38] (Table 1).

### 2.2. Animal Surgery and Tissue Sample Collection

Animal surgery was performed as previously described in Inserra et al. [36]. Briefly, animals were anesthetized with ketamine chlorhydrate 13.5 mg/kg body weight (Holliday Scott S.A., Buenos Aires, Argentina) and xilacine clorhydrate 0.6 mg/kg body weight (Laboratorios Richmond, Buenos Aires, Argentina). Blood samples taken by puncture of the inferior vena cava were centrifuged at 3000 rpm for 15 min, and the serum was separated, aliquoted, and stored at −20°C for the subsequent hormonal assays. After bleeding, animals were sacrificed by an intracardiac injection of Euthanyle 0.5 ml/kg body weight (sodic pentobarbital, sodic diphenyl hydantoinate; Brouwer S.A., Buenos Aires, Argentina). Pituitaries and ovaries were surgically removed and either stored at −80°C for subsequent PCR studies or fixed for 48 h in cold 4% paraformaldehyde (PFA) in 0.01 M phosphate-buffered saline (PBS, pH 7.4) for histological studies.

All experimental protocols performed in the present study were reviewed and authorized by the Institutional Committee on the Use and Care of Experimental Animals of Universidad Maimonides, Argentina. Handling and sacrifice of animals were performed in accordance with all local, state, and federal guidelines for the care and utilization of laboratory animals. Husbandry of the animals met the National Institutes of Health Guidelines for the Care and Use of Laboratory Animals [39].

### 2.3. Immunohistochemistry (IH) and Immunofluorescence (IF) with Confocal Microscopy

PFA-fixed ovary and anterior pituitary of each animal were dehydrated through a graded series of ethanol and embedded in paraffin. Each gland was sectioned at 5 *μ*m thick and mounted onto coated slides. To analyze pituitary PRL immunoreactivity, 3 slides with 3 adenohypophyseal sections per slide corresponding to rostral end, medial, and caudal end regions were tested (5 animals per experimental group). Three distinct adenohypophyseal fields at each section were analyzed avoiding superposition among them. For each ovarian marker (PRLR, LHR, and 3*β*-HSD), 3 slides (containing 3 tissue sections per slide) corresponding to anterior, middle, and posterior regions of the ovary were tested and the immunoreactivity was analyzed in all the corpora lutea. Adjacent slides were tested for each marker (5 animals per experimental group). Briefly, sections were subjected to antigen retrieval by boiling sections in 10 mM sodium citrate buffer pH 6.0 for 20 min. Endogenous peroxidase activity was quenched, and nonspecific binding sites for immunoglobulins were blocked by incubating sections with 10% normal serum. Immunoreactivity was detected by incubating sections overnight in a humid chamber at 4°C with the primary antibody (Table 1, supplementary material). Specificity of primary antibodies was corroborated in adjacent sections by omission of the primary antibodies.

Immunoreactivity was revealed with a biotinylated goat anti-rabbit IgG or rabbit anti-goat IgG, as appropriated, followed by incubation with avidin-biotin complex (ABC Vectastain Elite kit, Vector Laboratories, Burlingame, USA). The reaction was visualized with 3,3′-diaminobenzidine (DAB kit, Vector Laboratories, Burlingame, USA). Sections were counterstained with hematoxylin for morphological orientation, dehydrated, and mounted. Sections were imaged using an optic microscope (BX40, Olympus Optical Corporation, Tokyo, Japan) fitted with a digital camera (390CU 3.2 Mega Pixel CCD Camera, Micrometrics, Spain).

In order to evaluate qualitatively the differences in the levels of immunoreactivity of each marker among the experimental groups, an immunohistochemical scoring combining the percentage of positive cells and the stain intensity was performed (“−” = negative, “+” = weak, “++” = moderate, and “+++” = strong reactivity).

For colocalization of PRLR/3*β*-HSD and of PRLR/LHR, anti-PRLR-, anti-LHR-, and anti-3*β*-HSD-specific antibodies were employed (Table 1, supplementary material). PRLR/3*β*-HSD: antibodies were incubated overnight and revealed with Alexa-Fluor 488 coupled horse anti-rabbit IgG for anti-PRLR IgG detection and Alexa-Fluor 555 coupled donkey anti-mouse IgG for anti-3*β*-HSD. PRLR/LHR: after an overnight incubation with anti-PRLR IgG, the tissue sections were revealed with Alexa-Fluor 555 coupled horse anti-rabbit IgG and then incubated overnight with anti-LHR IgG and revealed with Alexa-Fluor 488 coupled horse anti-rabbit IgG. Both fluorescent-coupled antibodies were purchased at Invitrogen Corp. (Invitrogen, California, USA) and used at a 1 : 500 dilution. Finally, slides were mounted with Vectashield (Vector Laboratories, Burlingame, USA). In order to eliminate any emission crosstalk between the fluorophores, sequential line scanning (lambda strobing mode) was used when acquiring the images. Control sections using each single antibody were also developed and scanned by the three lasers to verify that emission was detected only in the specific single channel. Five animals were tested per group.

### 2.4. RNA Isolation

For isolation of total RNA, each piece of tissue (ovaries and pituitary glands) was homogenized with TRIzol (Invitrogen, California, USA) according to the manufacturer's instructions. RNA concentration was quantified by absorption at 260 nm (GeneQuant, Amersham Biosciences, England). One *μ*g of total RNA was treated with 1 *μ*l DNaseI (Invitrogen, California, USA) in 1 *μ*l 10X DNase Reaction Buffer (Invitrogen, California, USA) for 30 minutes at 37°C, and the reaction was stopped with 1 *μ*l EDTA 50 mM (Invitrogen, California, USA) for 10 minutes at 65°C. The RNA was reverse transcribed into first-strand cDNA using 1.5 *μ*l random hexamer primers 50 *μ*M (Applied Biosystems, California, USA), 200 U reverse transcriptase (RevertAid™ M-MuLV, Fermentas, Massachusetts, USA), 4 *μ*l first strand buffer 5x (Fermentas, Massachusetts, USA), 2 *μ*l dNTP mixture 10 mM (Invitrogen, California, USA), and 0.5 *μ*l RNase inhibitor (Ribolock™, Fermentas, Massachusetts, USA), at a 20 *μ*l final volume reaction. The reverse transcriptase was omitted in control reactions, where the absence of PCR-amplified DNA indicated the isolation of RNA free of genomic DNA. The reaction was carried out at 72°C for 10 minutes and 42°C for 60 minutes and stopped by heating at 70°C for 10 minutes. cDNA was stored at −20°C until use.

### 2.5. Real-Time Semiquantitative Polymerase Chain Reaction (qPCR)

The abundances of pituitary PRL and ovarian PRLR, LHR, 3*ß*-HSD, and 20*α*-HSD transcripts of each experimental group were determined by qPCR. Gene-specific primer sets for PRL, PRLR, 3*ß*-HSD, and 20*α*-HSD were customized (Life Technologies, California, USA), whereas for LHR and GAPDH, primers already described in the literature were used. Primer sequences are shown in Supplementary Table 2.

For the analysis of ovarian PRLR gene expression, we designed specific primers over highly homologous regions obtained from the multiple alignments of PRLR mRNA sequences from several species evolutionarily related to vizcachas (*Cavia porcellus*, *Chinchilla lanigera*, *Mus musculus*, *Octodon degus*, and *Homo sapiens*) using the Clustal Omega software (http://www.ebi.ac.uk/Tools/msa/clustalo/). We successfully amplified PRLR in the pituitary, mammary gland, ovary, and liver of vizcachas (not shown), and the sequencing of ovarian PRLR exhibited an 88% homology with chinchilla (*Chinchilla lanigera*); an 85% with guinea pig (*Cavia porcellus*); an 82% with the desert mole rat (*Heterocephalus glaber*); a 78% with the Damara mole rat (*Fukomys damarensis*), the tree shrew (*Tupaia chinensis*), the ground squirrel (*Ictidomys tridecemlineatus*), and the norwegian rat (*Rattus norvegicus*); a 77% with mice (*Mus musculus*) and degu (*Octodon degus*); and a 74% with humans.

For PRL primer design, we proceeded in the same fashion as we did for PRLR, focusing on those highly conserved PRL mRNA sequences among the evolutionarily related species mentioned above. PRL primers were tested using cDNA from different vizcachas' tissues; pituitary and mammary glands revealed PRL transcription but not the liver nor the ovaries (not shown). Sequencing the PCR product of pituitary PRL of *L. maximus* showed an 88% homology with the PRL mRNA of the chinchilla; an 86% with the desert mole rat; an 85% with the Damara mole rat, the guinea pig, and the degu; a 77% with the wild boar (*Sus scrofa*); and a 75% with primates (*Gorilla gorilla*, *Pan troglodytes*, and *Homo sapiens*).

Amplification reactions were carried out using the SYBR Green PCR Master Mix (Applied Biosystems, California, USA) on a Stratagene MPX500 cycler (Stratagene, California, USA). After an initial denaturation step (95°C for 10 min), 40 cycles of a 2-step amplification protocol (95°C for 15 s, 60°C for 45 s) were completed. Melting curve analysis was performed to verify the amplification specificity. Relative quantification of gene expression was performed according to the mathematical model of Pfaffl [40]. Briefly, the expression ratio was determined for each sample by calculating (E_target_)^∆Cq(target^)/(E_GAPDH_) ^∆Cq(GAPDH)^, where E is the efficiency of the primer set and ∆Cq (quantification cycle) is the difference in the threshold cycle with ∆Cq = Cq _(normalization cDNA)_ − Cq _(experimental cDNA)_. The amplification efficiency of each primer set was calculated from the slope of a standard amplification curve of log (ng cDNA) per reaction versus Cq value (*E* = 10^−(1/slope)^). Efficiencies of 2.0 ± 0.1 were considered optimal. Five animals were tested per experimental group, and each sample was analyzed in duplicate along with nontemplate controls. Each target gene expression was normalized to that of GAPDH. To confirm PRL, PRLR, 3*ß*-HSD, and 20*α*-HSD identities, purified PCR products (MinElute Gel Extraction kit, Qiagen, Hilden, Germany) were sequenced with a 3130xl Genetic Analyzer (Applied Biosystems, California, USA) by the Genomic Unit of the Biotechnology Institute, Instituto Nacional de Tecnología Agropecuaria (INTA), Buenos Aires, Argentina.

### 2.6. Enzyme-Linked Immunosorbent Assay (ELISA) for P4

Serum P4 level was determined by ELISA using an EIAgen Progesterone kit (Adaltis S.r.l., Rome, Italy) as previously described by our laboratory [29]. Briefly, a direct solid phase enzyme immunoassay detecting a range of 0.18–40.0 ng/ml (0.48–127.2 nmol/l) was developed. The absorbance of the solution measured at 450 nm was inversely related to the concentration of P4 in the sample. Calculation of P4 level was made by reference to a calibration curve. All captured vizcachas were evaluated, and their P4 levels are depicted in Table 1.

### 2.7. Radioimmunoassay (RIA) for LH

Serum LH level was determined by RIA with kits from the National Hormone and Pituitary Program, National Institute of Diabetes, Digestive and Kidney Diseases, USA, as previously described by our laboratory [36]. Results were expressed in terms of rat LH standards using the following standards: for LH iodination: r-LH-I10, reference preparation rat LH-RP-3, (AFP7187B) and anti-rat LH-S11 (AFPC697071P) [41]. Assay sensitivity was 0.31 ng/ml. Intra- and interassay coefficients of variation were 7.2 and 11.4%, respectively. All captured vizcachas were evaluated (Table 1). Pooled pituitary homogenates of high LH levels were serially diluted to prepare a standard curve for the vizcachas, and the parallelism with the rat standard curve was confirmed, as previously described [28]. All captured vizcachas were evaluated, and their P4 levels are depicted in Table 1.

### 2.8. Statistical Analysis

Values were expressed as the mean ± standard error of the mean. Statistical differences were determined by a one-way analysis of variance (ANOVA) and Newman-Keuls multiple comparison post hoc tests using Prism 4.0 (GraphPad Software Inc., San Diego, California, USA). Differences were considered statistically significant when *p* < 0.05. A correlation analysis among PRLR, LHR, and 3-HSD was performed using the nonparametric Kendall rank correlation coefficient [42].

## 3. Results

### 3.1. Ovarian Expression of PRLR and LHR

**Figure 1:**
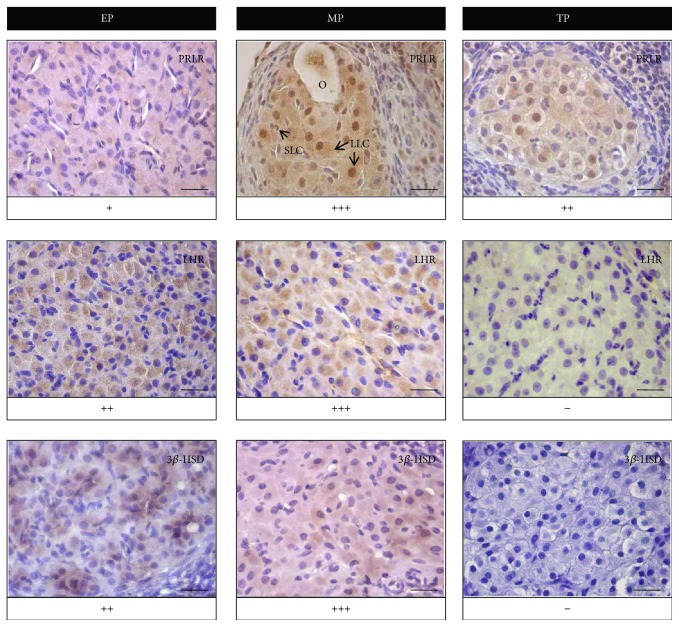
Luteal levels of PRLR, LHR, and 3*ß*-HSD increased in pregnant-ovulating vizcachas. Representative photomicrographs of luteal cells in ovary cross sections immunostained for PRLR, LHR, and 3*ß*-HSD, at early pregnancy (EP), midpregnancy (MP), and term pregnancy (TP) of vizcachas. Immunoreactivity is shown in brown and nuclei counterstained with hematoxylin. Immunohistochemical scoring was determined using a 4-point scale: “+” = weak, “++” = moderate, “+++” = strong, and “−” = negative reactivity. LLC: large luteal cells; SLC: small luteal cells; O: nonovulated oocyte into a secondary corpus luteum. Scale bar represents 25 *μ*m.

**Figure 2:**
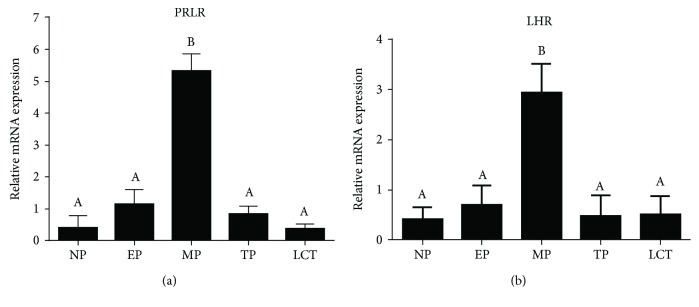
Transcription of ovarian PRLR and LHR sharply increased in pregnant-ovulating vizcachas. qPCR analysis of mRNA abundance in ovaries revealed that both PRLR (a) and LHR (b) exhibit their highest levels of transcripts at MP. Values expressed as the mean ± SEM. Significant differences were determined by a one-way ANOVA (*p* < 0.05, *n* = 5 per group). Different letters indicate significant differences. GAPDH was used as housekeeping gene. (NP) non-ovulating non-pregnant, (EP) early pregnant, (MP) midpregnant, (TP) term pregnant, and (LCT) lactating nonpregnant groups.

We examined the expression of PRLR and LHR by immunohistochemistry in corpora lutea and by qPCR in the whole ovaries of adult vizcachas throughout the experimental reproductive stages (Figures 1 and 2). Since the ovaries of the nonpregnant groups of this study (LCT and NP) exhibited almost the total absence of corpora lutea, the luteal immunoreactivity was analyzed only at the stages of pregnancy.

We detected PRLR immunoexpression throughout the entire gestation. Such a positive mark had a grainy appearance and was evidenced in both small and large luteal cells (Figure 1). PRLR immunoreactivity was weak at the beginning of pregnancy; it became markedly stronger at midgestation and decreased again near parturition time. Some PRLR reactivity was also observed in nuclei at both mid- and term pregnancies.

Once the specificity of PRLR-customized primers was verified as described in Material and Methods, PRLR mRNA levels were examined in ovaries of vizcachas throughout the reproductive stages. Analysis of qPCR data showed a 4-fold rise when transitioning from early pregnancy to midpregnancy and a marked decline at the end of gestation (*p* < 0.05, *n* = 5 per group) (Figure 2(a)).

We studied ovarian LHR expression as a key marker of luteinization (Figure 1). We detected LHR-positive reactivity in luteal cells along the three stages of pregnancy. This mark had grainy appearance as well. LHR exhibited its most intense immunoreactivity during midpregnancy and then fell to almost imperceptible levels in the term pregnant group.

For the analysis of LHR by qPCR, we used specific primers previously validated for vizcachas by Fraunhoffer et al., [37] (Table 2, supplementary material). Ovarian LHR transcription levels along the reproductive stages of vizcachas exhibited a quite similar expression pattern during gestation than that determined by immunohistochemistry in corpora lutea. Ovarian LHR mRNA level was significantly higher at midpregnancy, the reproductive stage characterized by the pseudoovulatory event (*p* < 0.05, *n* = 5 per group) (Figure 2(b)). At term pregnancy and during nonpregnant stages (NP and LCT), LHR transcription remained low.

### 3.2. Ovarian Steroidogenic Enzymes 3*ß*-HSD and 20*α*-HSD

**Figure 3:**
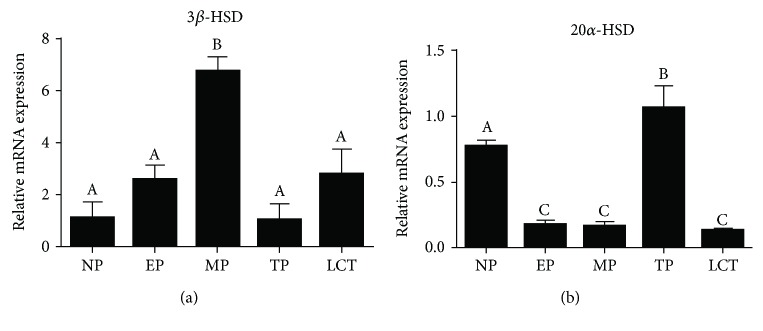
Transcription of ovarian 3*ß*-HSD and 20*α*-HSD exhibited opposite patterns during midgestation and term gestation. qPCR analysis of 3*ß*-HSD mRNA abundance revealed the highest levels at MP (a) while 20*α*-HSD transcription remained low (b). Such relation was reversed at term pregnancy, when 20*α*-HSD mRNA levels reached its maximum and 3*ß*-HSD mRNA abundance dropped low close to the recorded basal values (NP). Values expressed as the mean ± SEM. Significant differences were determined by a one-way ANOVA (*p* < 0.05, *n* = 5 per group). Different letters indicate significant differences. GAPDH was used as housekeeping gene. (NP) non-ovulating non-pregnant, (EP) early pregnant, (MP) midpregnant, (TP) term pregnant, and (LCT) lactating nonpregnant groups.

In order to examine the expression of both enzymes involved in P4 metabolism, 3*ß*-HSD and 20*α*-HSD, we first customized primers for PCR (Table 2, supplementary material). We analyzed both ovarian transcripts profiles throughout the reproductive stages of adult female vizcachas (Figure 3).

At early pregnancy, 3*β*-HSD ovarian expression was slightly higher than that of non-ovulating non-pregnant group, exhibited its highest level at midpregnancy, almost tripling the values of the early pregnant group, and then, it drastically dropped to a sixth before parturition (TP). During lactation, 3*β*-HSD expression levels did not differ from those of the previous reproductive stage (*p* < 0.05, *n* = 5 per group) (Figure 3(a)). In addition, luteal 3*β*-HSD immunohistochemical scoring analysis showed high reactivity during early pregnancy and midpregnancy but a complete absence at the end of pregnancy (Figure 1).

Instead, 20*α*-HSD gene expression remained low during early pregnancy, almost undetectable at midpregnancy, and sharply increased 5-fold at the end of gestation (TP). After parturition and while females were lactating their litters (LCT), ovarian 20*α*-HSD expression significantly dropped to levels similar to those recorded before the pseudoovulatory event. Non-ovulating non-pregnant group showed significantly lower levels than midpregnant but higher than the remaining experimental groups (*p* < 0.05, *n* = 5 per group) (Figure 3(b)).

Given the similarity of the mRNA expression profiles, we performed a correlation analysis of 3*ß*-HSD, PRLR, and LHR transcription levels using the nonparametric test based on a rank correlation known as Kendall's rank correlation coefficient or Kendall's tau coefficient (*τ*). The results showed that *τ* = 1 for all 5 analyzed reproductive stages (*p* < 0.05, *n* = 5) which indicates an identical correlation among 3*ß*-HSD, PRLR, and LHR for each reproductive stage.

### 3.3. Colocalization of PRLR and 3*ß*-HSD

**Figure 4:**
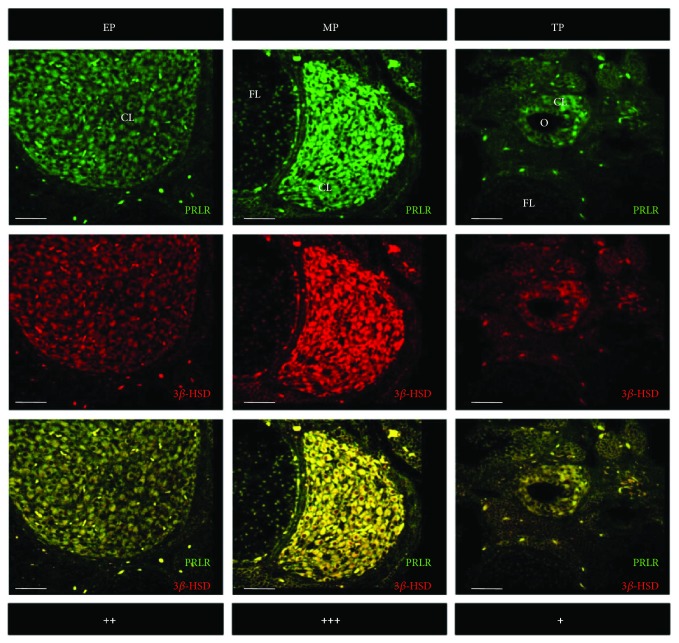
Strongest colocalization of PRLR and 3*ß*-HSD occurred in luteal cells of pregnant-ovulating vizcachas. Representative confocal photomicrographs of ovary sections of pregnant vizcachas stained for PRLR (green) and 3*ß*-HSD (red) using double-labeled immunofluorescence. Yellow stained represents coexpression of PRLR and 3*ß*-HSD. Scale score: “+” = weak, “++” = moderate, and “+++” = strong. Scale bar: 50 *μ*m. CL: corpus luteum; FL: follicle; O: nonovulated oocyte into a secondary corpus luteum. EP, MP, and TP stand for early pregnancy, midpregnancy, and term pregnancy, respectively.

**Figure 5:**
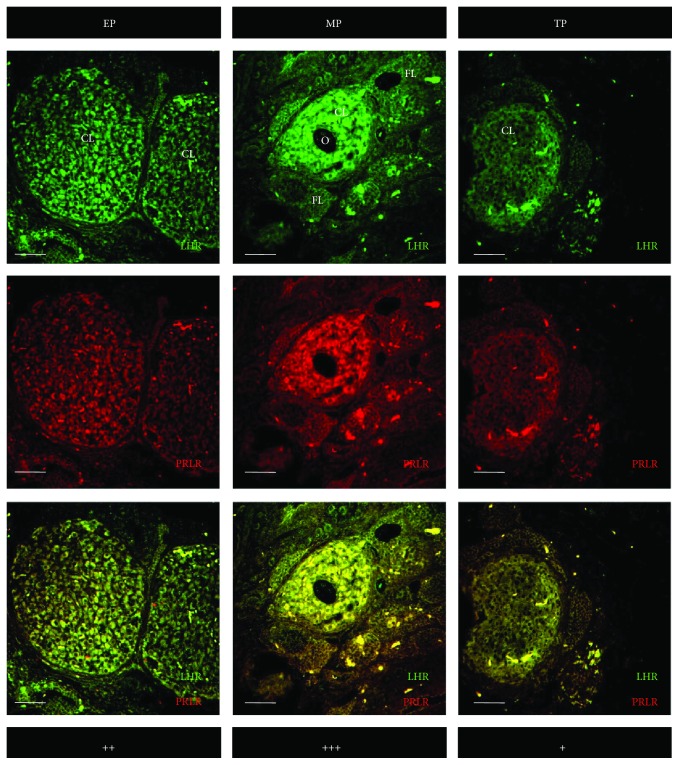
Strongest colocalization of PRLR and LHR occurred in luteal cells of pregnant-ovulating vizcachas. Representative confocal photomicrographs of ovary sections of pregnant vizcachas stained for PRLR (red) and 3*ß*-HSD (green) using double-labeled immunofluorescence. Yellow stained represents coexpression of PRLR and LHR. Scale score: “+” = weak, “++” = moderate, and “+++” = strong. Scale bar: 50 *μ*m. CL: corpus luteum; FL: follicle; O: nonovulated oocyte into a secondary corpus luteum. EP, MP, and TP stand for early pregnancy, midpregnancy, and term pregnancy, respectively.

Figure 4 depicts the colocalization of PRLR and 3*ß*-HSD using double-labeled immunofluorescence in corpora lutea of representative vizcachas throughout gestation. PRLR and 3*ß*-HSD were observed in both small and large luteal cells. A qualitative estimation indicated that the maximum colocalization of PRLR and 3*ß*-HSD occurred during midpregnancy, and this result is consistent with what was analyzed for each marker separately both by immunohistochemistry and by qPCR. Moreover, PRLR colocalized with LHR as well, throughout the 3 analyzed pregnancy stages, being midpregnancy the stage depicting maximum expression of both receptors (Figure 5).

### 3.4. PRL Levels

**Figure 6:**
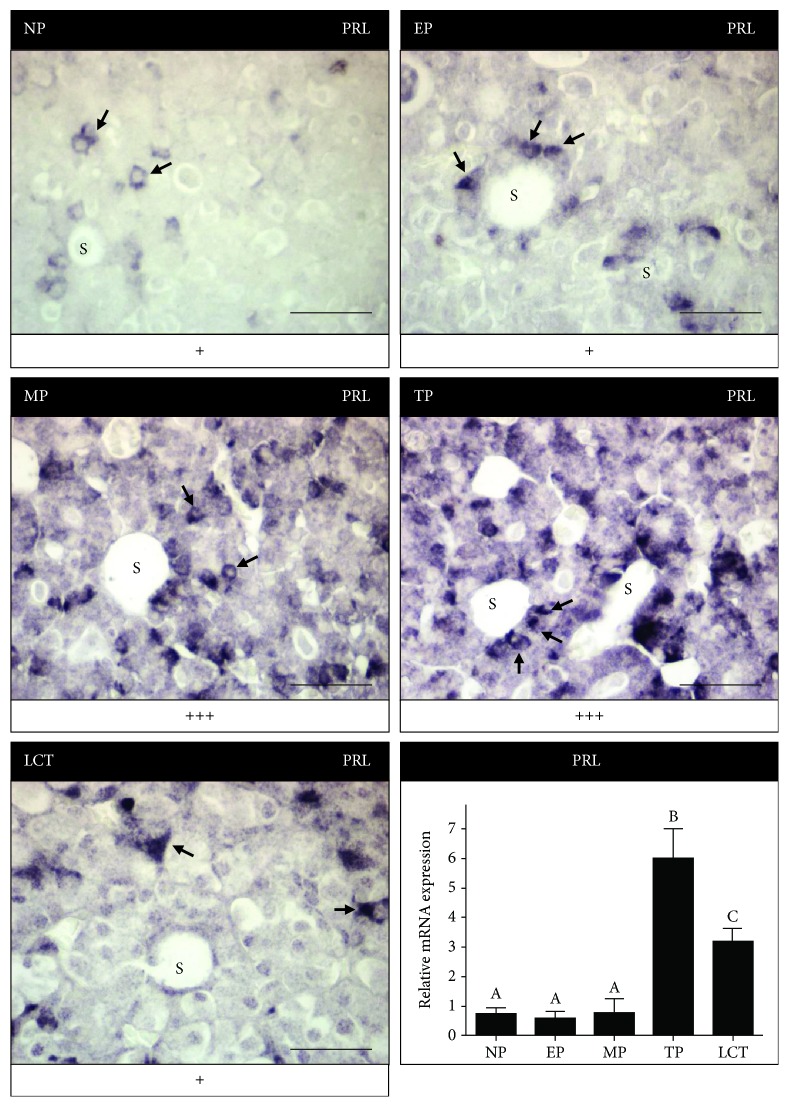
Pituitary expression of PRL increased during pregnancy. Representative photomicrographs of pituitary gland cross sections of adult non-ovulating non-pregnant (NP), early pregnant (EP), midpregnant (MP), term pregnant (TP), and lactating non-pregnant (LCT) vizcachas immunostained for PRL. Immunoreactivity is shown in black (color modification of diaminobenzidine precipitation by addition of nickel) and is highlighted by arrows. “**s**” indicates a capillary sinusoidal space. Scale score: “+” = weak, “++” = moderate, and “+++” = strong. Scale bar represents 50 *μ*m. Bottom right qPCR analysis of pituitary PRL mRNA abundance revealed that TP group exhibits the highest level of transcripts. After parturition and while nurturing the litter (LCT), PRL mRNA quantity decreases although is still significantly higher than NP, EP, and MP (*p* < 0.0005, *n* = 5 per group, one-way ANOVA). Different letters indicate significant differences. GAPDH was used as housekeeping gene.

We attempted to analyze circulating PRL levels by both radioimmunoassay (RIA) and enzyme-linked immunosorbent assay (ELISA). Regrettably, neither of these techniques proved to be successful for PRL serum determination in vizcachas. Therefore, to have an approximated idea of the PRL production along the reproductive stages of adult female vizcachas, we examined its expression in the pituitary by immunohistochemistry and by qPCR (Figure 6).

We detected PRL immunoexpression in the pituitary gland pars distalis in all the experimental groups (Figure 6). Immunoexpression was mainly observed in the cytoplasm of isolated lactotrophs or lactotropic cells forming follicular structures. Non-ovulating non-pregnant females showed a weak reactivity considered as the basal production of PRL (Figure 6). The immunoexpression of PRL increased in mid- and term pregnant groups, exhibiting in the latter the highest reactivity, especially in those lactotropic cells closest to the capillaries. During lactation, pituitary PRL immunoexpression decreased to values similar to the basal production measured in non-ovulating non-pregnant animals (Figure 6). Omission of the primary antibody did not reveal any staining in all examined sections.

Once the specificity of PRL-customized primers was established (see Material and Methods), we evaluated the variations of PRL gene transcription in the pituitary gland of *L. maximus* among the five reproductive stages. The results obtained from qPCR also indicated a sharp rise of PRL at the end of gestation (TP). However, unlike what we had measured for the pituitary protein expression by immunohistochemistry, PRL mRNA levels remained significantly high during lactation (*p* < 0.0005, *n* = 5 per group) (Figure 6).

## 4. Discussion

P4 is essential for the development of normal pregnancy, and inadequate production of this steroid from the corpus luteum is associated with pregnancy loss ([43] for a review). In the present work, we showed for the first time, a comparative relation between the expressions of several molecules directly or indirectly involved in the synthesis and secretion of luteal P4 throughout the reproductive cycle of female vizcachas. Previous reports have shown that *Lagostomus maximus* displays a peculiar P4 profile during gestation, which is characterized by two well-defined stages: a first wave that begins to decline after approximately 70 days of pregnancy (d.p.), reaching a minimum around 100 d.p., and a second increase at 110–120 d.p., with a final decline at parturition time. It is likely that high levels of P4 in the first stage are linked to the activity of the primary corpora lutea, whereas the increase during the second stage is largely a result of what is secreted by the newly formed corpora lutea in the pseudoovulatory event at the end of the third month of pregnancy [31, 37].

Here, we found that the ovarian PRLR expression profile and the P4-circulating levels follow a concordant pattern along the reproductive cycle of adult female vizcachas. Maximum expression of PRLR recorded at midpregnancy coincides with the peak of serum P4, while the decrease of this steroid recorded right before parturition is accompanied by a drastic decrease in both PRLR immunoreactivity and transcript levels. Moreover, the expression of ovarian 3*ß*-HSD, the enzyme responsible for the conversion of pregnenolone in P4, also follows the expression pattern of PRLR throughout the reproductive cycle of *L. maximus*, tripling its values when going from early pregnancy to midpregnancy and dropping them to a sixth at the end of gestation. Although luteal 3*ß*-HSD is considered to be constitutively expressed throughout pregnancy, several reports have shown that 3*ß*-HSD can be regulated by PRL and gonadotropins [3, 44, 45].

Our analysis of LHR expression in the ovary of vizcachas revealed an active transcription level at midpregnancy and in response to the LH surge. Such an increase followed by an abrupt fall of LHR mRNA towards the end of pregnancy could be indicating a receptor desensitization mechanism. In fact, previous reports have shown that a LHR desensitization of luteal cells upon ligand-binding results in a decline in steady state levels of LHR mRNA [46, 47]. Such loss of LHR mRNA during receptor downregulation is due to an increased degradation of the receptor mRNA rather that to an inhibition of transcription [3, 48].

Activation of LHR in follicular cells by the LH surge causes ovulation and luteinization. This process alters their responsiveness to external signals allowing luteal cells to respond to a new set of hormones, the most important being PRL and LH [3]. Midpregnant vizcachas exhibited high levels of transcription for PRLR as well, which could be indicative of a cross talk mechanism between these two hormones for a coordinated dialogue towards an accurate luteinization process in *L. maximus* as previously documented for mice [22]. It has been shown that the positive regulation of the expression of LHR during CL formation in conventional rodent models is modulated by PRL [49–51]. Therefore, it is worth noting that the Kendall's *τ* calculation confirmed a high correlation between the expression profile of the ovarian LHR gene and that of PRLR/3*β*-HSD during vizcacha's gestation. This result is in agreement with the high colocalization of PRLR with both LHR and 3*β*-HSD detected in luteal cells of midgestating animals, which suggests a possible role of PRL during pseudoovulation and luteinization of *L. maximus*.

Interestingly, our data showed that ovarian 20*α*-HSD, the enzyme that catalyzes the conversion of P4 into its inactive form, is repressed during pregnancy of vizcachas, except at term gestation, when it rises dramatically. Moreover, the expression of 20*α*-HSD along gestation contrasted with both the 3*ß*-HSD and the PRLR/LHR profiles.

There have been several reports showing that ovarian 20*α*-HSD expression is modulated by PRL. Deficient signal transducers and activators of transcription (Stats) Stat5a/Stat5b mice have shown that the impairment of these major PRL signaling mediators trigger pregnancy loss that correlates with an increased 20*α*-HSD and a decreased serum P4 [52]. In addition, PRLR knockout mice exhibit corpora lutea undergoing regression and insufficient levels of P4 to support implantation due to the absence of downregulation of 20*α*-HSD by PRL [21]. Even more, administration of P4 in the form of subcutaneous pellets rescues preimplantation embryo development and implantation in PRLR knockout females [53]. Another interesting report by Clementi and coworkers [54] also suggests that the luteotropic effect of the decidual tissue in rats is mediated through the secretion of pituitary PRL, which in turn stimulates P4 biosynthesis by increasing luteal 3*β*-HSD activity, and inhibits P4 catabolism by diminishing luteal 20*α*-HSD activity.

In the present work, we estimated variation of PRL levels throughout the reproductive cycle of vizcachas by measuring pituitary PRL expression by immunohistochemistry and mRNA levels by qPCR. So far, our attempts to determine circulating PRL levels either by RIA or by ELISA did not yield positive results probably due to a subject linked to the specificity of the antibodies used in both techniques. PRL immunodetection was evidenced in the cytoplasm of lactotropic cells localized in the pars distalis as previously described by Filippa and Mohamed [55]. During gestation, particularly from midpregnancy, we detected a significant accumulation of pituitary PRL that reached its maximum at term pregnancy. The rate of PRL transcription increased along pregnancy as well. PRL drives the process of lactation, and thus it would be expected to find at this stage the highest level of this hormone in the pituitary gland. Yet, we measured a marked decrease of PRL immunoreactivity in pituitary of lactating compared to term-pregnant females. A possible explanation of this decrement would be that, during lactation, the constant demand exerted by the litter through the stimulus of suckling may cause the newly synthesized hormone to be immediately poured into the circulatory system, preventing its accumulation into the lactotrophs.

The pituitary PRL profile here characterized clearly has a different pattern than its ovarian receptor. However, it must be considered that mammary glands are the primary target of PRL. In fact, pituitary PRL expression determined in this study is in agreement with the documented increased expression of PRLR in mammary glands of vizcachas throughout gestation and this is tightly related to the mammary gland preparation for the eventual nurturing of the forthcoming offspring [29, 38, 56].

In short, if the luteal steroidogenesis of pregnant vizcachas is, as we hypothesized, modulated in part by PRL, our results suggest that such regulation would be driven by the different levels of PRLR expressed in the ovaries at each stage of pregnancy.

This is the first attempt to relate PRL as well as its ovarian receptor to some key steroidogenic markers involved in the production of P4 during gestation of vizcachas. Notwithstanding the present investigation is a static study and therefore a causal relationship between PRLR/LHR increased expression and the increment in P4 levels cannot be ascertained, it is an interesting start point to inquire into the mechanisms that drive luteal steroidogenesis in *L. maximus*. This takes special relevance if we consider that for other rodents such as mice and rats, PRLR is a key component regulating ovarian function and governing the regulation of P4 secretion [2, 23, 57].

In summary, the results of our investigation showed that the maximal expression recorded for PRLR, LHR, and 3*β*-HSD occurs at midpregnancy, when the pseudoovulatory event occurs, and coincides with both a minimal 20*α*-HSD expression and a marked increase in P4 serum levels. Instead, the period of time preceding parturition is characterized by 20*α*-HSD in its highest level of expression, which contrasts with the minimum of PRLR/LHR/3*β*-HSD and with the lowest levels of circulating P4. The expression patterns of these two steroidogenic enzymes suggest that PRL and LH through its ovarian receptors would indirectly favor luteal P4 production in early and midpregnant vizcachas by both stimulating 3*β*-HSD and by negatively modulating 20*α*-HSD, whereas such modulation would invert at the end of pregnancy allowing the fall of P4 that ultimately elicits parturition in *L. maximus.* Our results suggest an active role of PRL as an LH partner in the modulation of luteal steroidogenesis and P4 production throughout gestation of vizcachas.

## Figures and Tables

**Table 1:** Inclusion criteria for the experimental groups.

	Time of capture	Number of embryos in the uterus	Crown-heel length of foetuses (mm)	Estimated gestational age (number of days)^a^	Ovulatory stigmata	Serum LH (ng/ml)	Serum P4 (ng/ml)
Early pregnant (EP)	April	8–12	10^b^	25–35	No	0.62 ± 0.2	5.71 ± 2.8
Midpregnant (MP)	July	1–2	90–115	90–110	Yes	3.20 ± 0.7	14.52 ± 3.95
Term pregnant (TP)	August	1–2	145–156	144–154	No	1.21 ± 0.18	0.75 ± 0.43
Lactating nonpregnant (LCT)	September	—	—	—	No	0.12 ± 0.1^c^	0.21 ± 0.1
Non-ovulating non-pregnant (NP)	September	—	—	—	No	0.02 ± 0.01^c^	0.77 ± 0.4

^a^According to [37]. ^b^Size of implantation sites (mm). ^c^Values below the detection limits of the assay.

## Supplementary Materials

Supplementary Materials Supplementary Table 1: list of primary antibodies used for immunohistochemistry and confocal colocalization. Supplementary Table 2: list of primer sequences used for the qPCR. Conditions for qPCR reaction are explained in detail in the Material and Methods section.
