# Supplementary material for: Prolactin Is a Strong Candidate for the Regulation of Luteal Steroidogenesis in Vizcachas (Lagostomus maximus)
Source: Int J Endocrinol. 2018 Jun 14;2018:1910672. doi: 10.1155/2018/1910672 (PMC6022330; doi:10.1155/2018/1910672)
Supplement: Supplementary Materials — Supplementary Table 1: list of primary antibodies used for immunohistochemistry and confocal colocalization. Supplementary Table 2: list of primer sequences used for the qPCR. Conditions for qPCR reaction are explained in detail in the Material and Methods section. [file 1910672.f1.doc]

Table 1: List of primary antibodies

| **Antibody** | **Brand** | **Dilution** | **References** |
| --- | --- | --- | --- |
| Rabbit polyclonal anti-**PRLR** | H-300, sc-20992, Santa Cruz Biotechnology Inc. | 1:200 | Halperin *et al.*, 2013 |
| Rabbit polyclonal anti-**LHR** | H-50, sc-25828, Santa Cruz Biotechnology Inc. | 1:200 | Fraunhoffer *et al.*, 2017 |
| Rabbit polyclonal anti-**PRL** | A0569, Dako | 1:200 | Filippa and Mohamed, 2010 |
| Goat polyclonal anti-**3ß-HSD** | P-18, sc-30820, Santa Cruz Biotechnology Inc. | 1:100 | Peralta *et al.*, 2016 |

Table 2: Primers sequences.

| Gene | Primer sequences | Product size  (base pairs) | validation |
| --- | --- | --- | --- |
| **PRL** | F: 5’ GTCAAACCTGCTCCTGTGC 3’  R: 5’GAGCAGGCCCAGCAAATTC3’ | 257 | costumized for the present work |
| **PRLR** | F: 5’GCCAGATCATGGGTACTGGAG3’  R:5’ CTTTCCACCAATTCCTGGGCC3’ | 195 | costumized for the present work |
| **LHR** | F: 5’CCACCAAATTGCAGGCCCT3’  R: 5’CAGTGGCTRGGGTAGGTC3’ | 145 | Fraunhoffer *et al.*,2017 |
| **3ß-HSD** | F: 5’GGACAAGGCCTTCAGACCAG3’  R: 5’GAGATGCCTTGGCAGGCTC3’ | 126 | costumized for the present work |
| **20α-HSD** | F: 5’GCAACCAGGTAGAATGCCA3’  R: 5’TGGTAGCGAAGGGCAATC3’ | 218 | costumized for the present work |
| **GAPDH** | F: 5’CCAGAACATCATCCCTGCAT3’  R: 5’GTTCAGCTCTGGGATGACCTT3’ | 67 | Gonzalez *et al*., 2012 |
